# Isolated Schwannoma of the Upper Eyelid Margin in a 50-year-old Male

**DOI:** 10.4274/tjo.26032

**Published:** 2016-12-01

**Authors:** Mehmet Serdar Dervişoğulları, Yüksel Totan, Ümran Yıldırım

**Affiliations:** 1 Medicana Konya Hospital, Ophthalmology Clinic, Konya, Turkey; 2 Maya Eye Disease Center, Ankara, Turkey; 3 Düzce University Training and Research Hospital, Pathology Clinic, Düzce, Turkey

**Keywords:** Eyelid margin, Histopathology, schwannoma

## Abstract

Schwannomas (neurilemmomas) are benign neurogenic tumours of peripheral nerves. They originate from Schwann cells, which form the neural sheath. Although Schwannomas and neurofibromas are the most common primary peripheral nerve tumours, Schwannomas are rarely observed in ophthalmic areas. When they occur, ocular Schwannomas are usually located in the orbit, uveal tract and conjunctiva. Isolated eyelid Schwannomas are reported infrequently. Herein, we describe a case of eyelid Schwannoma in a 50-year-old man. The diagnosis of Schwannoma was made after the eyelid mass was removed by excisional biopsy, so this entity should be included in the differential diagnosis of eyelid margin tumours.

## INTRODUCTION

Schwannomas (neurilemmomas) are benign tumours derived from the Schwann cells of the peripheral nerve sheath. The tumour is a solitary mass that can be located in soft tissues throughout the body. It has a smooth surface and grows slowly. It is mostly asymptomatic and may occur at any age or gender in the general population. Multiple neurofibromas are a distinctive feature of neurofibromatosis (NF) type 1 and bilateral acoustic Schwannomas are a feature of NF type 2. Because of their tendency to occur in spinal nerve roots, sympathetic nerves, cervical nerves and vagus nerves, Schwannomas are mostly seen in the head and neck.^1^ They occasionally arise in the orbit and infrequently in the conjunctiva1, uveal tract^2^ and sclera.^3^ Eyelid Schwannomas, especially at the eyelid margin, are uncommon; only 2 cases in adults have been reported to date.

## CASE REPORT

A 50-year-old man was referred to us with a history of a painless nodule that had enlarged slowly on his right upper eyelid for 2 years. He had no history of NF or any other nodules. Ocular examination was normal but there was a firm, non-tender nodule measuring 3x4x4 mm in the lateral side of the right upper eyelid margin. Clinical findings of NF were not observed. The lesion was thought to be a papilloma and was completely removed by shave excision under local anesthesia.

**Figure 1:**
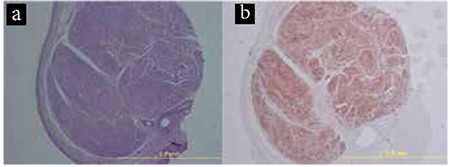
Histologic analysis of an eyelid margin Schwannoma from a 50-year-old male, spindle cells arranged in a palisading fashion in an Antoni A area (Hematoxylin-eosin, x40) (a) S100 positivity in spindle cells (x40) (b)

Pathological studies showed a mass approximately 3 mm in diameter on macroscopic examination. On microscopic examination, histopathologic bundles of spindle cells with no mitotic activity (Figure 1a) were observed. No histopathologic features of malignancy were present. Immunohistochemical analysis revealed a strong positive reaction for S100 protein (Figure 1b). Tumour cells did not react with spinal musculoskeletal atrophy, Desmin or CD34. The final diagnosis was benign Schwannoma of the eyelid margin.

**Figure 2:**
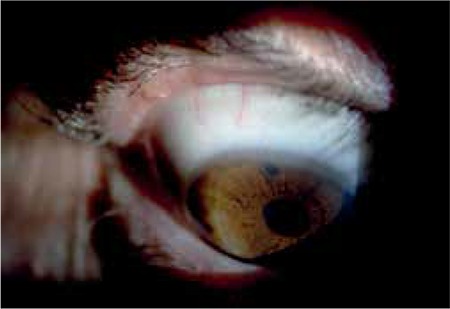
No recurrence was observed 1 year after excision of eyelid margin Schwannoma

The patient was asymptomatic and there were no symptoms or signs of recurrence one year later (Figure 2).

## DISCUSSION

Proliferating Schwann cells of peripheral nerve sheaths form Schwannoma (or neurilemmoma). It is a rare, slow-growing, benign, asymptomatic neoplasm and may occur in any myelinated peripheral or cranial nerve. They occasionally arise in the orbit and infrequently in the conjunctiva1, uveal tract2 and sclera.^3^ The reported origins for orbital Schwannomas are oculomotor, ciliary and supraorbital nerves. Eyelid Schwannomas are presumed to originate from supraorbital, supratrochlear and infraorbital nerves. Schwannomas typically manifest as a single benign neoplasm. Multiple Schwannomas in one patient is usually indicative of NF. In Schwannomatosis (neurilemmomatosis), multiple non-vestibular Schwannomas are observed with no other stigmatas of NF type 1 or NF type 2.^4^ Clinico-pathologic variants of Schwannoma include conventional Schwannoma, cellular Schwannoma, and melanotic Schwannoma.^5^ Microscopically, they may demonstrate a biphasic pattern, and areas of highly cellular (Antoni type A) and myxoid matrix (Antoni type B) may be observed.^5^ Degenerative changes may occur in time.^6^ Prognosis is poor if the cells are fusiform, contain melanin granules, or if epithelioid cells are present.^7^ Nevertheless, malignant transformation has not been reported in eyelid Schwannomas and total excision seems to be curative. The most important feature for diagnosis of a Schwannoma is still its strong reactivity to S100 protein in immunochemistry.^1,2,3,4,5,6,7^

Schwannoma of the eyelid margin in adults was first reported in 2007 by Lopez-Tizon et al.^8^ The second report was in 2012, by Cheng et al.^9^ The first reported case of an eyelid margin Schwannoma was a slowly enlarging 0.4 cm nodule, thought to be an inclusion cyst on the right upper eyelid margin for 1 year, which did not recur for 12 years after pentagonal full-thickness excision. The second report was a 35-year-old man who presented with a translucent, painless, cyst-like nodule with a smooth surface located on the right lower eyelid margin, resembling hidrocystoma and treated by shave excision.

Our patient had isolated eyelid Schwannoma with no family history or clinical findings of NF. The mass was located on the lateral half of the eyelid margin and the tumor probably arose from branches of the supraorbital nerve. Schwannomas are rare tumours that can occur in unusual locations, including the eyelid margin, and should be considered in the differential diagnosis of the eyelid margin tumours. Complete surgical excision is necessary to avoid recurrence. Incomplete removal is associated with eventual recurrence and more aggressive behavior.^5,6^ The lesion was thought to be a papilloma and shave biopsy was performed. Because histologic diagnosis was Schwannoma, the patient was planned to be followed up closely for any sign of recurrence. There was no recurrence in 3-months follow-up. Malign transformation has not been reported with eyelid margin Schwannomas.

Schwannomas of ophthalmic interest are rare but may mimic inclusion cysts or chalazia.^8,10,11^ They are extremely uncommon at the eyelid margin but should be considered in the differential diagnosis of any solid eyelid margin lesion.

### Ethics

Informed Consent: It was taken.

Peer-review: Externally peer-reviewed.
